# Plasma Markers of Oxidative Stress in Patients with Gestational Diabetes Mellitus in the Second and Third Trimester

**DOI:** 10.1155/2016/3865454

**Published:** 2016-10-10

**Authors:** Hongwei Li, Qian Yin, Ning Li, Zhenbo Ouyang, Mei Zhong

**Affiliations:** ^1^Department of Obstetrics & Gynecology, Nanfang Hospital, Southern Medical University, Guangzhou, China; ^2^Department of Obstetrics & Gynecology, Puyang Oilfield General Hospital, Puyang, China; ^3^Department of Gynecology, Guangdong No. 2 Provincial People's Hospital, Guangzhou, China

## Abstract

*Objective.* To determine plasma markers of oxidative stress during the second and third trimester of pregnancy in patients with gestational diabetes mellitus (GDM).* Study Design.* We conducted a prospective nested case-control study involving 400 pregnant women, 22 of whom developed GDM. As control group, 30 normal pregnant women were chosen randomly. Plasma samples were analyzed for 8-iso-prostaglandin F2*α* (8-iso-PGF2*α*), advanced oxidative protein products (AOPPs), protein carbonyl (PCO), glutathione peroxidase-3 (GPX-3), and paraoxonase-1 (PON1) at 16–20 weeks, 24–28 weeks, and 32–36 weeks of gestation.* Results.* Compared to control subjects, the plasma levels of PCO, AOPPs, and 8-iso-PGF2*α* were elevated at 16–20 weeks' and 32–36 weeks' gestation in GDM. There was no significant difference in PCO and 8-iso-PGF2*α* at 24–28 weeks in GDM. GPX-3 was statistically significantly increased at 16–20 weeks and 32–36 weeks in GDM. PON1 reduced in patients with GDM. No significant differences were found at 24–28 and 32–36 weeks between the GDM and control groups. In GDM, PCO, AOPPs, and 8-iso-PGF2*α* levels were higher and GPX-3 and PON1 levels were lower in the second than the third trimester.* Conclusion.* Oxidation status increased in GDM, especially protein oxidation, which may contribute to the pathogenesis of GDM.

## 1. Introduction

Gestational diabetes mellitus (GDM) is an idiopathic disease that occurs during pregnancy. Women with GDM have a high risk of developing type 2 diabetes, metabolic syndrome, and cardiovascular disease. The prevalence of metabolic syndrome in women with International Association of Diabetes in Pregnancy Study Group- (IADPSG-) defined GDM is three times greater than in women with normal glucose tolerance during pregnancy [1]. Gunderson et al. showed that history of GDM may be a useful marker of early atherosclerosis independent of prepregnancy obesity in women who have not developed type 2 diabetes or the metabolic syndrome [2]. The Hyperglycemia and Adverse Pregnancy Outcome (HAPO) study demonstrated that high maternal blood glucose correlates with increasing fetal morbidity and mortality [3]. The offspring of diabetic mothers are also at high risk of metabolic syndrome and diabetes mellitus in childhood and adulthood [4, 5]. The exact pathogenesis of GDM is uncertain. Clarifying the pathogenic mechanism is important for early diagnosis and treatment and is helpful in improving maternal and infant prognoses.

Recently, attention has been focused on the association between oxidative stress and GDM. It has been clarified that patients with type 2 diabetes mellitus have severe oxidative stress [6]. Some studies have shown enhanced oxidation products in patients with GDM and reduced antioxidant capacity, suggesting that oxidative stress may contribute to the development and progression of GDM [7–11]. However, the relation between the different levels of various plasma oxidative markers and the development of GDM during pregnancy has not been systematically characterized.

Lipid peroxidation can reflect the level of oxidative damage, which results in damage of the cell membranes. The products of lipid oxidative damage have important roles in various physiological and pathological conditions. It is widely recognized that proteins are the main original targets for oxidative damage. An experimental study indicated that protein oxidation precedes the oxidative damage of lipids and may represent an independent mechanism of cellular damage in addition to membrane lipid peroxidation [12]. In type 2 diabetes mellitus, the markers of oxidative lipid and protein damage are significantly enhanced compared to those of normal individuals and are even higher in those with diabetic complications [13–15], showing that oxidative lipid and protein damage may contribute to microvascular and macrovascular complications.

A complex and integrated antioxidant system plays a crucial role in protecting cells or tissues from damage as the result of reactive oxygen species (ROS). The expression and activity of antioxidants are changed during oxidative stress. Decreased antioxidant levels have been found in patients with type 2 diabetes mellitus and its complications [13, 16, 17]. However, there are discrepancies with regard to the antioxidative defense in various diseases.

The aim of this study was to investigate the oxidative stress status during the second and third trimester of pregnancy in patients with GDM by determining plasma levels of 8-iso-prostaglandin F2*α* (8-iso-PGF2*α*) as a marker of lipid peroxidation, advanced oxidative protein products (AOPPs) and protein carbonyl (PCO) as markers of protein oxidation, and plasma glutathione peroxidase-3 (GPX-3) and paraoxonase (PON1) as markers of antioxidative defense, to explore the role of oxidative stress in the development and progression of GDM.

## 2. Materials and Methods

This was a prospective nested case-control study. A total of 400 pregnant women receiving prenatal care before 20 weeks' gestation at the obstetric clinic of the Nanfang Hospital were recruited between March 2012 and November 2012. Inclusion criteria were as follows: Han ethnicity; singleton pregnancy; being of the age of 18 to 40 years; BMI < 40 Kg/m^2^; no smoking or drinking; no prepregnancy diabetes or family history of diabetes; absence of hypertension and cardiovascular and other systemic disease; no infection or inflammation; no history of abnormal pregnancy. During follow-up, women who experienced abortion, premature delivery, or stillbirth; had preeclampsia, premature rupture of membranes, and other pregnancy complications, or did not deliver at Nanfang Hospital were excluded. The study was approved by the Ethics Committee of Nanfang Hospital, and informed consent was obtained from all subjects. There were 22 women who developed GDM. After diagnosis, GDM subjects were treated with dietary therapy and more rigorous glucose monitoring. One of the 22 women diagnosed with GDM was given insulin therapy because glucose did not achieve the target level with only dietary therapy. We randomly chose 30 normal pregnant women as a control group.

Fasting blood glucose was measured (FBG < 7.0 mmol/L) after a period of fasting, usually at least eight hours without food or liquid (except water) in the morning during the first prenatal visit. All subjects had a 75 g oral glucose tolerance test at 24 to 28 weeks' gestation and were screened for GDM according to the IADPSG criteria (plasma glucose thresholds: fasting 5.1 mmol/L [92 mg/dL], 1 h 10.0 mmol/L [180 mg/dL], and 2 h 8.5 mmol/L [103 mg/dL]) [18].

### 2.1. Blood Sampling

Venous blood samples were drawn after a 12-hour overnight fast at 16–20 weeks, 24–28 weeks, and 32–36 weeks of gestation. The samples were centrifuged within 30 min at 3000 ×g, 4°C for 15 min. Plasma samples were stored at −80°C until being assayed.

### 2.2. Assay of Plasma AOPP Levels

Plasma AOPP levels were measured by spectrophotometric assay [19]. The plasma was diluted 1 : 10 with phosphate buffered saline (PBS), and 10 *μ*L of diluted sample, 200 *μ*L of PBS (the blank), and 200 *μ*L of chloramine-T standard solution (0–100 *μ*mol/L) were placed in wells of a 96-well microliter plate. Ten microliters of 1.16 M potassium iodide was added, followed by 10 *μ*L of acetic acid. The absorbance was read immediately at 340 nm. The concentration of AOPPs was expressed in chloramine-T equivalents (*μ*mol/L).

### 2.3. Assay of Plasma PCO Levels

Plasma levels of PCO were determined by sandwich enzyme-linked immunosorbent assay using a commercial kit (RUIJI, Shanghai, China) according to the manufacturer's directions. The interassay and intra-assay coefficients of variation were 10%, respectively.

### 2.4. Assay of Plasma 8-Iso-PGF2*α*, GPX-3, and PON1 Levels

The 8-iso-PGF2*α*, GPX-3, and PON1 levels were determined by sandwich enzyme-linked immunosorbent assay using a commercial kit (CUSABIO, Wuhan, China) according to the manufacturer's directions. The interassay and intra-assay coefficients of variation were less than 8% and 10%, respectively.

### 2.5. Statistical Analysis

The data were analyzed with SPSS 13.0 software (SPSS, Chicago, IL). All continuous variables were presented as mean ± standard deviation (SD). Differences between the GDM group and control group were analyzed using the independent *t*-test and the normal distribution of data was checked. A repeated measures ANCOVA was used to compare differences between any two gestational ages in the GDM group. Correlations between quantitative variables were evaluated using Pearson's or Spearman's correlation coefficients. *P* < 0.05 was considered as statistically significant.

## 3. Results

As shown in Table 1, there was no significant difference between the groups in maternal age, BMI, gravidity/parity, triglycerides (TG), cholesterol, low-density lipoprotein (LDL), high-density lipoprotein (HDL), mean HbA_1C_, and fasting glucose. Compared with the control group, 1 h glucose and 2 h glucose were significantly increased in patients with GDM (*P* < 0.05).

Levels of antioxidative enzymes in the plasma of patients with GDM are shown in Table 2. The activity of GPX-3 was statistically significantly increased at 16–20 weeks and 32–36 weeks of gestation in GDM patients when compared to control subjects (both *P* < 0.05). There was no significant difference at 24–28 weeks' gestation (*P* = 0.23). Plasma PON1 was reduced in patients with GDM. However, no significant differences between the groups were found at 24–28 (*P* = 0.35) and 32–36 weeks (*P* = 0.56).

Concentrations of plasma products of oxidative stress were increased. Plasma AOPPs at different gestational ages were significantly higher in the GDM group compared to the control group (*P* < 0.05). Levels of plasma PCO (*P* = 0.12) and 8-iso-PGF2*α* (*P* = 0.09) were increased in patients with GDM compared to those of the control group, although there was no significant difference at 24–28 weeks.

**Figure 1:**
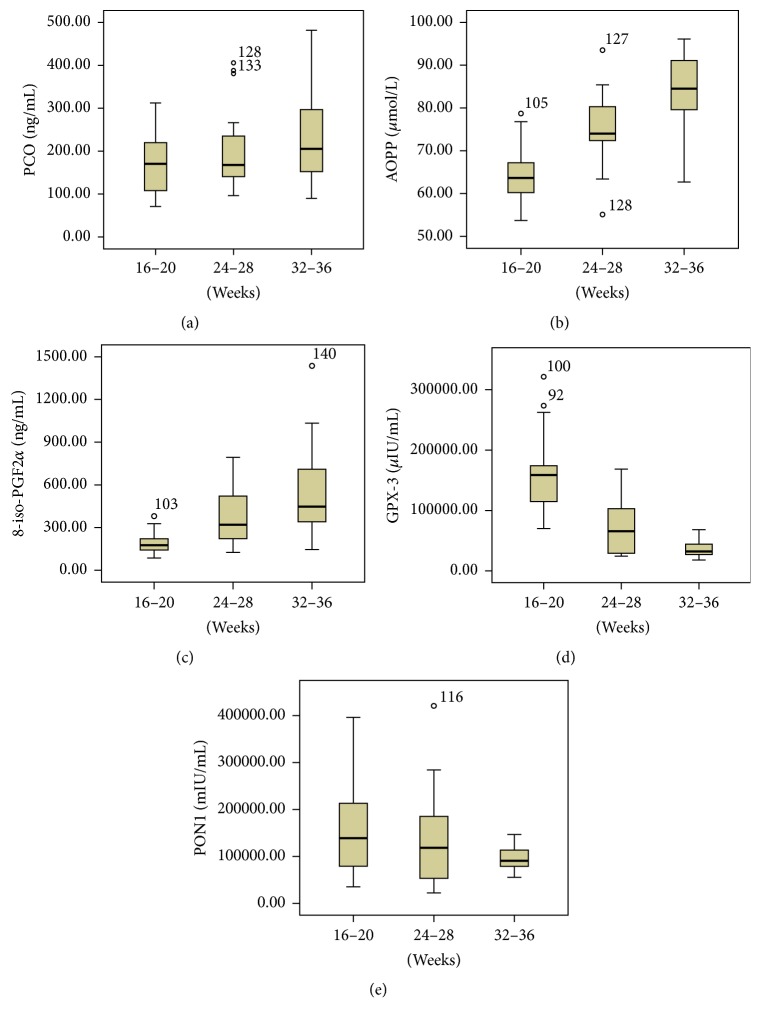
The level of PCO (a), AOPP (b), 8-iso-PGF2*α* (c), GPX-3 (d), and PON1 (e) at 16–20 weeks, at 24–28 weeks, and at 32–36 weeks in GDM.

In the GDM group, plasma AOPPs were higher at 24–28 weeks than at 16–20 weeks (*P* < 0.05), and AOPPs and plasma 8-iso-PGF2*α* were higher at 32–36 weeks than at 24–28 weeks (both *P* < 0.05). Increased PCO levels were found at 32–36 weeks compared to levels at 16–20 weeks (*P* < 0.05). However, no significant difference was found between 24–28 weeks and 16–20 weeks and the same between 32–36 weeks and 24–28 weeks. On the other hand, GPX-3 was decreased at 24–28 weeks compared to 16–20 weeks (*P* < 0.05) and lower at 32–36 weeks than at 24–28 weeks. The activity of plasma PON1 was lower at 32–36 weeks than at 16–20 weeks and 24–28 weeks (*P* < 0.05) and was decreased at 24–28 weeks compared to 16–20 weeks and was decreased at 32–36 weeks compared to 24–28 weeks, but there were no significant differences (Figure 1).

Correlation analysis was performed to assess the association between variables of oxidative stress and glucose and HbA_1C_ in the GDM group. The plasma level of AOPPs at 16–20 weeks was positively correlated with the OGTT 1 h glucose level and HbA_1C_ (*R* = 0.435, *P* < 0.05; *R* = 0.655, *P* = 0.00, resp.). Moreover, the AOPP level at 24–28 weeks was positively correlated with the OGTT 1 h and 2 h glucose level and HbA_1C_ (*R* = 0.529, *P* < 0.05; *R* = 0.524, *P* < 0.05; *R* = 0.546, *P* < 0.01, resp.). At 32–36 weeks, there was a significant relationship between AOPPs and the fasting glucose level (*R* = 0.670, *P* = 0.00). Plasma PCO at 16–20 weeks was positively correlated with the OGTT 1 h glucose level (*R* = 0.444, *P* < 0.05).

**Figure 2:**
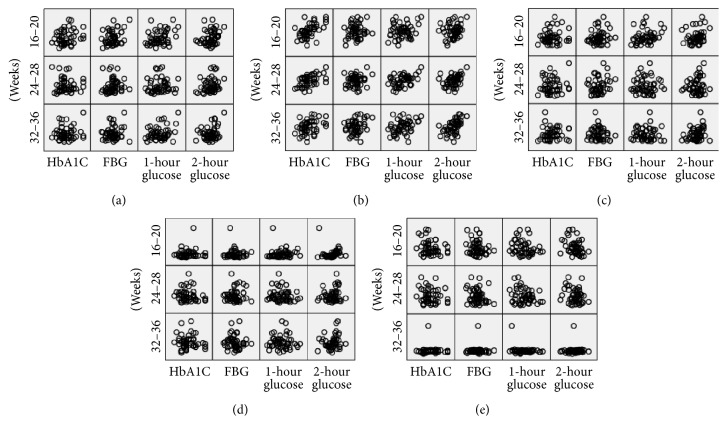
Relationships between PCO (a), AOPP (b), 8-iso-PGF2*α* (c), GPX-3 (d), PON1 (e), and A1C and OGTT glucose level in GDM. FBG: fasting blood glucose.

While plasma GPX-3 at 24–28 weeks was negatively correlated with the OGTT 1 h glucose level (*R* = −0.441, *P* < 0.05), at 32–36 weeks, a significant negative correlation was found between the GPX-3 level and HbA_1C_ (*R* = −0.429, *P* < 0.05). There was no significant correlation between 8-iso-PGF2*α* and glucose level or PON1 and HbA_1C_. No correlation was found between AOPPs, PCO, 8-iso-PGF2*α*, GPX-3, and PON1 (Figure 2).

## 4. Discussion

Recently, the role of oxidative stress in GDM has attracted the attention of investigators. However, the relation between the different levels of various plasma oxidative markers and the development of GDM during pregnancy has not been systematically characterized. In the present study, higher levels of oxidative stress markers were found in patients with GDM than in normal pregnant women. We found that markers of oxidative stress were increased and antioxidants were decreased with the progress of gestation in GDM, suggesting that there was increased oxidative protein and lipid damage and that the oxidation status was increased with the progression of gestation in GDM.

F2-isoprostanes are a family of prostaglandin isomers produced by peroxidation of cell-membrane phospholipids or circulating LDL [20]. 8-iso-PGF2*α* is considered to be a sensitive and stable biomarker of lipid peroxidation. There was a significant increase in 8-iso-PGF2*α* in patients with type 1 diabetes mellitus compared with the control group [21, 22]. It has been reported that levels of 8-iso-PGF2*α* are significantly higher in serum or urine in diabetic patients than in healthy control subjects, and a positive correlation was found between 8-iso-PGF2*α* and both fasting glucose and HbA_1C_ in diabetic patients with vascular complications [14, 23]. An experiment in rats showed that 24 h urinary excretion of 8-iso-PGF2*α* was increased in diabetic rats compared to a normal control group, and this difference was more marked in pregnancy [24]. Moreover, an increased 8-iso-PGF2*α* level has been found in patients with both types of diabetes, suggesting that it may be a useful marker for assessing the association between lipid oxidation damage and hyperglycemia. 8-iso-PGF2*α* from placenta, adipose tissue, and skeletal muscle is greater in women with GDM than in healthy pregnant women [25, 26].

AOPPs are the final products of various protein oxidation formed by oxidative stress and are considered novel markers of oxidative protein damage. Elevation of the AOPPs level has been found to play an important role in many diseases. Increased AOPPs may be an independent risk factor for atherosclerotic disease [27]. Numerous studies indicate that AOPPs are significantly increased in patients with type 2 diabetes mellitus and its complications, and AOPPs have been recognized as useful markers to estimate the degree of oxidative protein damage [28, 29]. A clinical study has found that AOPPs could serve as an early marker of vasculopathy in individuals with type 2 diabetes [30]. Karacay et al. reported that circulating levels of AOPPs were increased at 24–36 weeks of gestation in GDM comparing to normal pregnancies [31].

PCO is a sensitive, stable marker of oxidant-mediated protein damage and is the most widely used. There is evidence of a close relationship between PCO and impaired glycemic control in type 2 diabetes mellitus [32]. Gelisgen et al. conducted a study of 23 women with GDM and 22 women without GDM and determined that plasma AOPPs and PCO levels were significantly increased [8].

In our study, elevated concentrations of 8-iso-PGF2*α*, AOPPs, and PCO were observed, suggesting that oxidative damage may be enhanced in patients with GDM compared to normal subjects. Moreover, we found that 8-iso-PGF2*α*, AOPPs, and PCO levels were significantly increased at 16–20 weeks, before diagnosis of GDM, suggesting that increased oxidative stress may occur before the onset of GDM and increases with the progression of gestation. We speculate that increased oxidative stress may contribute to the development and progression of GDM.

PON1 is an antioxidant enzyme that can protect LDL and HDL from oxidation, but also it plays a key antiatherosclerotic role. Decreased PON1 has been found in patients with type 2 diabetes and diabetic macrovascular complications [33, 34]. Compared to normal pregnant women, the activity of PON1 was decreased in patients with GDM, and it has been shown that reduced PON1 may be due to increased plasma protein oxidative damage [8, 35]. In our study, plasma PON1 levels were lower in patients with GDM compared with the control group, although there was no significant difference at 24–28 weeks and 32–36 weeks.

GPX-3 is the only extracellular isoform of the glutathione peroxidase family and is also a major antioxidative enzyme. The data reported in a former study on GPX-3 levels in different diseases remain controversial. A number of studies have shown that GPX-3 levels are decreased in women with papillary serous ovarian cancer in a stage-dependent manner and also found decrease in women with gastric cancer [36, 37]. However, there was an increase in serum GPX-3 in overweight and obese subjects [38]. An experimental study revealed that expression of the GPX-3 gene was increased in the hearts of diabetic mice and concluded that increased GPX-3 may play a significant role in protecting cardiomyocytes from oxidative stress [39]. Conversely, in our study the patients with GDM were found to have an increase of GPX-3 in plasma. We think that the increase of GPX-3 in patients with well-controlled GDM may be described as compensation for the excessive generation of ROS.

In our study, AOPPs and PCO levels were positively correlated with HbA_1C_ and the OGTT 1 h glucose level at 16–20 weeks in the GDM group. Moreover, we found that AOPPs at 24–28 weeks had a more significant positive correlation with HbA_1C_ and the OGTT glucose level. Additionally, a negative correlation was only found between GPX-3 and the OGTT 1 h glucose and HbA_1C_ at 24–28 weeks. There was no correlation between PON1, 8-iso-PGF2*α*, HbA_1C_, and the OGTT glucose level. According to the results of correlation analyses, we concluded that protein oxidation may play a key role in impaired glycemic equilibrium in GDM. We speculate that the measurement of protein oxidation markers may have a certain predictive value for GDM.

Normal human pregnancy is considered as a state of enhanced oxidative stress, while pathologic pregnancies, including GDM, are associated with a heightened level of oxidative stress, owing to both overproduction of free radicals and a defect in the antioxidant defenses [40], which has also been validated by our study. However, no systematic research on the relation between plasma oxidative markers in different gestation and the development or the progression of GDM has been done before. Our team focused on the fluctuation of these markers in plasma during pregnancy and tried to confirm the association with GDM. In the following investigation, we will look deep into the detailed mechanism under GDM, especially how the oxidative stress works on the pathogenesis of GDM.

There are some limitations in the present study. First, because of time constraints and capital limitations, the number of study subjects was small. Second, the study was limited to the second and third trimester of gestation, with no follow-up either in the first trimester of gestation or postpartum. Third, the study did not analyze the difference between subjects with good glycemic control and those with poor glycemic control because there were only a few cases of poor glycemic control in the GDM group.

## 5. Conclusions

In conclusion, increased lipid peroxidation and protein oxidative damage in GDM patients compared to normal pregnant women was found before the onset of GDM. With the progression of gestation, the oxidation status was enhanced in GDM. There was a close association between protein oxidation and impaired glucose metabolism. The expression of antioxidants in plasma in GDM was altered, but we cannot evaluate the overall tendency of antioxidative defense. We can conclude that oxidative stress is enhanced in GDM. Increased oxidative stress, especially protein oxidation, may contribute to the pathogenesis of GDM and may have predictive value. Further studies with a larger sample size should be performed to confirm the role of oxidative stress in GDM.

## Figures and Tables

**Table 1:** Clinical and metabolic characteristics of study subjects.

	GDM (*n* = 22)	Controls (*n* = 30)	*P* value
Maternal age, years	28.82 ± 0.67	27.37 ± 0.70	0.15
BMI before gestation, Kg/m^2^	20.98 ± 0.51	19.74 ± 0.42	0.07
Gravidity	2.18 ± 0.27	1.64 ± 0.19	0.10
Parity	1.14 ± 0.14	1.20 ± 0.20	0.82
Triglycerides (mg/dL)			
16–20 weeks	1.71 ± 0.11	1.77 ± 0.13	0.72
24–28 weeks	2.10 ± 0.12	2.32 ± 0.12	0.21
32–36 weeks	3.33 ± 0.24	3.29 ± 0.14	0.88
Total cholesterol (mg/dL)			
16–20 weeks	5.54 ± 0.16	5.31 ± 0.13	0.28
24–28 weeks	6.39 ± 0.20	6.38 ± 0.16	0.97
32–36 weeks	6.33 ± 0.29	6.18 ± 0.24	0.68
LDL (mg/dL)			
16–20 weeks	2.89 ± 0.17	2.82 ± 0.10	0.69
24–28 weeks	3.28 ± 0.16	3.35 ± 0.19	0.79
32–36 weeks	3.56 ± 0.21	3.15 ± 0.17	0.14
HDL (mg/dL)			
16–20 weeks	1.98 ± 0.08	1.91 ± 0.06	0.50
24–28 weeks	2.02 ± 0.06	1.88 ± 0.05	0.62
32–36 weeks	1.94 ± 0.11	2.07 ± 0.06	0.26
HbA_1C_ (%)	5.15 ± 0.69	5.02 ± 0.05	0.11
OGTT fasting glucose (mmol/L)	4.25 ± 0.09	4.10 ± 0.08	0.22
OGTT 1-hour glucose (mmol/L)	8.94 ± 0.32	7.35 ± 0.25	0.00^*∗*^
OGTT 2-hour glucose (mmol/L)	9.01 ± 0.16	6.81 ± 0.22	0.00^*∗*^

Data are mean ± SD.  ^*∗*^ *P* < 0.001.

**Table 2:** Markers of oxidative stress and antioxidants in women with GDM and without GDM (controls).

	GDM			Controls		
	16–20 weeks	24–28 weeks	32–36 weeks	16–20 weeks	24–28 weeks	32–36 weeks
PCO (ng/mL)	172.77 ± 13.59^*∗*^	200.77 ± 19.23	232.48 ± 21.93^*∗*^	136.82 ± 10.05	169.09 ± 13.10	180.81 ± 11.57
AOPPs (*μ*mol/L)	64.74 ± 1.39^*∗*^	74.92 ± 1.67^△^	83.59 ± 1.87^△^	59.98 ± 1.54	65.83 ± 1.57	72.86 ± 1.34
8-iso-PGF2*α* (ng/mL)	196.14 ± 16.78^*∗*^	356.96 ± 40.10	538.72 ± 67.36^*∗*^	154.82 ± 9.89	289.74 ± 22.68	381.07 ± 37.26
GPX-3 (*μ*IU/mL)	160.42*E*3 ± 13.76*E*3^*∗*^	68.29*E*3 ± 8.50*E*3	35.65*E*3 ± 2.90*E*3^#^	83.71*E*3 ± 25.58*E*3	44.590*E*3 ± 3.49*E*3	26.46*E*3 ± 1.46*E*3
PON1 (mIU/mL)	162.88*E*3 ± 22.12*E*3^*∗*^	136.18*E*3 ± 20.98*E*3	97.00*E*3 ± 5.51*E*3	260.20*E*3 ± 28.89*E*3	165.77*E*3 ± 18.92*E*3	115.59*E*3 ± 25.73*E*3

Data are means ± SD.  ^#^ *P* < 0.01 versus controls.  ^*∗*^ *P* < 0.05 versus controls.  ^△^ *P* < 0.001 versus controls. There was no significance in PCO (*P* = 0.12), 8-iso-PGF2*α* (*P* = 0.09), GPX-3 (*P* = 0.23), and PON1 (*P* = 0.35) in 24–28 weeks compared to the control group. Meanwhile, in 32–36 weeks, no significance was found in PON1 (*P* = 0.56) compared to the controls.
